# Use of Cognitive Behavioral Therapy and Token Economy to Alleviate Dysfunctional Behavior in Children with Attention-Deficit Hyperactivity Disorder

**DOI:** 10.3389/fpsyt.2015.00167

**Published:** 2015-11-25

**Authors:** Luzia Flavia Coelho, Deise Lima Fernandes Barbosa, Sueli Rizzutti, Mauro Muszkat, Orlando Francisco Amodeo Bueno, Monica Carolina Miranda

**Affiliations:** ^1^Psychobiology Department, Universidade Federal de São Paulo, São Paulo, Brazil

**Keywords:** attention deficit hyperactivity disorder, cognitive behavioral therapy, treatment, token economy, children

## Abstract

Medication has proved highly efficacious as a means of alleviating general symptoms of attention-deficit hyperactivity disorder (ADHD). However, many patients remain functionally impaired by inappropriate behavior. The present study analyzed the use of cognitive behavioral therapy (CBT) with the Token-Economy (TE) technique to alleviate problem behavior for 25 participants with ADHD, all children (19 boys, mean age 10.11) on long-term methylphenidate medication, who were given 20 CBT sessions with 10 weeks of TE introduced as of session 5. Their ten most acute problem behaviors were selected and written records kept. On weekdays, parents recorded each inappropriate behavior and provided a suitable model for their actions. At weekly sessions, problem behaviors were counted and incident-free participants rewarded with a token. To analyze improvement (less frequent problem behavior), a list of 11 behavioral categories was rated: inattention, impulsivity, hyperactivity, disorganization, disobeying rules and routines, poor self-care, verbal/physical aggression, low frustration tolerance, compulsive behavior, antisocial behavior, lacking in initiative and distraction. Two CBT specialists categorized behaviors and an ADHD specialist ruled on discrepancies. Statistical analyses used were Generalized Estimating Equations with Poisson distribution and autoregressive order correlation structure. In the course of the sessions, problematic behaviors decreased significantly in seven categories: impulsiveness, hyperactivity, disorganization, disobeying rules and routine, poor self-care, low frustration tolerance, compulsive behaviors, and antisocial behaviors. Caregiver attitudes to children’s inappropriate behavior were discussed and reshaped. As functional improvement was observed on applying TE for 10 weeks, this type of intervention may be useful as an auxiliary strategy combined with medication.

## Introduction

Attention Deficit and Hyperactivity Disorder (ADHD) has become one of the most extensively researched neurodevelopmental disorders over the last 30 years. It is unequivocally recognized as a neurobiological disorder that causes significant impact in daily life and prevalence among children and adolescents worldwide has been estimated at around 5.2% (1). ADHD presents in three types: ADHD/I (predominance of inattention), ADHD/H (hyperactive and impulsive) and ADHD/C (combined) with each subtype showing different specific impairments and responses to treatment (2).

Studies have noted that ADHD in the course of its development is associated with increased risk of poor school performance, exam failure, school transfers, conflictive relationships with family and colleagues, development of internalizing symptoms (anxiety, depression, low self-esteem) and externalizing symptoms (conduct problems, delinquency, early experimenting with and abuse of substances) (3). Given all these impairments, in addition to unfavorable prognosis in the absence of treatment, revised ADHD treatment guidelines have ranged from medication to behavioral treatments such as psychoeducation, behavioral therapy, and interventions in school or other settings (4).

Patients diagnosed with ADHD have increasingly been prescribed medications, particularly stimulants, which have among the most extensively researched treatments for this disorder. Numerous clinical trials have found core symptoms of hyperactivity, impulsivity, and inattention showing statistically significant improvement as well as better academic performance, relations with family and peers and behavioral problems, and medication may even diminish the risk of subsequent psychiatric comorbidities (5, 6).

However, other researchers have found that side effects mean that pharmacological intervention is not always acceptable for parents, children, or even some clinicians. Negative effects such as loss of appetite, weight loss, difficulty falling asleep, trembling, dry mouth, nausea, abdominal pain, low energy, irritability, diarrhea, muscle tension, grinding teeth, and rare but important cases of sudden death due to pre-existing hidden cardiovascular problems may prevent continued use of psychostimulants (7–9).

In addition, cultural and gender differences may influence decision making and adherence to treatment and one study found differences in ADHD-explanatory models used by African-American and Caucasian parents (10). Another study of treatment preferences shown by parents of ADHD children found that 70.5% of its sample showed a strong tendency toward avoiding medication. Avoiders were more often males from higher social-economic and educational levels than the medication-preferring group (11).

In many cases, even with pharmacological intervention, patients may continue to display significant functional impairment in behavioral terms, thus compromising quality of life for individuals with ADHD and their families (12). Therefore, in order to diminish these impairments and improve overall functioning, multimodal treatment approaches have been increasingly proposed (9, 13).

The “Multimodal Treatment Study of children with ADHD” a multicenter study based on a sample of 579 children with ADHD, testing four treatments group (medication alone, behavioral therapy alone, medication and behavioral therapy combined, and a community-care group), showed improvements but with varying degrees of changes in their symptoms. Results from combined treatment were no better than those from medication alone (14).

Fabiano et al. reported the first meta-analysis of size-effect of behavior modification treatments in individuals with ADHD. Their analysis showed that behavioral treatments were efficacious (effect size 0.74) and they suggested should be directed to publicizing interventions in order to refine and improve community, school and mental health service interventions (15).

The most recent meta-analysis of ADHD treatment evaluated several types of intervention for children and adolescents including behavior modification, neurofeedback therapy, multimodal psychosocial treatment, school-based programs, working memory training, parent training, and self-monitoring. Findings showed more evidence for behavior modification and neurofeedback interventions improving ADHD cardinal symptoms, behavior and performance on neuropsychological tests (16).

Various types of non-pharmacological child or teen-centered treatments have been extensively used. Cognitive behavioral therapy (CBT) was one of the most efficacious non-medicamentous treatments (17). A recent study evaluated CBT efficacy for ADHD management of 68 adolescents (18). Positive effects of treatment were observed for dosage, parental rating of adherence to pharmacotherapy, adolescents’ self-reported personal adjustment (e.g., self-esteem), parent, and teacher-reported scales for symptoms of inattention, school attendance, failing school-year promotion, family and academic functionality, teacher-reported relations with peers, academic progress, and self-esteem.

The token-economy (TE) system is one of the most widespread for managing and measuring dysfunctional behavior. Children’s parents and other caregivers keep a record of daily or weekly occurrence of target behaviors initially formulated together with the child or teen. For children with ADHD, some researchers have suggested printing out these predetermined behaviors (rules) on cards (19). This technique uses as consequence strategy awards of points or tokens for desirable behavior (positive contingency reinforcement) and may condition significant impairments to display of inappropriate behaviors such as withdrawal of a previously gained token (response cost). DuPaul et al. reported efficacious strategies for behavioral interventions applicable in the school setting using the TE system as a strategy that may lead to improved behavior for task completion, and found clinically significant improvements in task-related attention as well as productivity and accuracy of work done in class, especially when combined with the cost response system (20).

This type of tool has been showing efficacy in improving ADHD related symptoms. This study assessed the impact of a TE program, i.e., positive reinforcement associated with response cost for adolescents diagnosed with ADHD. The results showed significantly lower frequency of behaviors between pre- and post-treatment in the school classroom context (21).

Another study evaluated the effects of stimulant medication plus TE technique on attention and disruptive behavior of three children diagnosed with ADHD during sports/games periods. The results showed that the TE was more effective than medication in terms of improving all three participants’ attentive behavior during sports/games. In addition, there was more improvement in attention when medication and TE were combined (22).

Although TE technique has been widely used for operant conditioning in children showing inappropriate and disruptive behavior (14–16, 23–30) cases presenting behavioral problems with lack of empathy, guilt, and self-monitoring difficulties (callous – unemotional) may present resistance and even behavioral deterioration if interventions involve cost of response or punishment. Interventions that maximize rewards with positive reinforcers appear to be most effective (31).

Brazilian and Latin American researchers have published very few studies on ADHD treatment combined with CBT or described the effect of operant conditioning techniques (TE) specifically for ADHD children. There is only one manual for the Brazilian population, which consists of 12 individual sections, but the efficacy of this model has not been tested so far (17).

In 2009, given the scarcity of CBT protocols for ADHD in Brazil, a major new ADHD intervention study set out to examine the effects of single and combined treatments (medication, CBT, attention training, working memory training). In this study, a 20-week CBT design was developed for group treatment to assess evidence of improvement and also because the group therapy model may be scaled for large numbers of patients, which would be beneficial for Brazil or other countries with similar health systems (32).

Fabiano et al. reported that the economy token “assigns points for targeted behaviors within the program setting,” and the technique is widely used in programs treating dysfunctional behaviors of various disorders (21, 22, 31, 33). However, most studies specifically comparing token economy technique effects have used counts of behaviors emitted as outcome measures, then comparing average frequencies at baseline date and post-intervention (21, 22, 31, 33). Other studies comparing effects of behavioral intervention have used standardized measures from cognitive tasks or behavioral inventories (14–16, 23, 24, 27, 28, 30). We are not aware of any that have specifically analyzed content or reduction of each individual’s inappropriate behavior (internalizing or externalizing) such as inattention, aggressive behavior, rule-breaking, or organization and planning issues.

Given the need for data on the effects of interventions based on functional difficulties, this study analyzed the effect of a 20-week CBT program using TE technique for a sample of 25 children and adolescents diagnosed with ADHD.

## Methods

### Participants

Children with ADHD were recruited from an outpatient clinic, associated with the Universidade Federal de São Paulo (UNIFESP), São Paulo, SP, Brazil, that specialized in the diagnostic of children and adolescents with neurodevelopmental disorders. Children were selected after parents/guardians reported symptoms of symptoms of agitation, being unable to remain quiet, and having difficulty paying attention. A screening interview conducted with a questionnaire covering developmental aspects and DSM-IV criteria. After this, they were submitted to neuropsychological and medical examinations (by a psychiatrist and a neurologist). The sample was recruited immediately after the diagnostic assessment, before subdividing into groups for intervention. Children with prematurity, diagnosis of other conditions (intellectual deficit, epilepsy, genetic syndromes, premature birth, HIV, hydrocephalus, brain lesions, etc.), current use of other medications except those used to treat ADHD, were excluded.

Caregivers of children were invited to complete the Socioeconomic Status Scale, this is a scale developed by the Brazilian Association of Companies and Research (www.abep.org), and assesses the level of education of the person responsible for the main income of the family and a total score on household comfort items. The scores are divided in eight different levels of socioeconomic status (classes ranging from A1 = very high socioeconomic status to E = very low socioeconomic status).

The final sample consisted of 25 children and adolescents diagnosed with ADHD (Table 1).

**Table 1:** **Demographic and participant characteristics**.

Variable	*M*	SD	%
Age (in years)	10.11	1.79	
Gender			76 (boys)
IQ	108.20	12.27	
Socioeconomic status			24 (A1–A2)
			36 (B1–B2)
			36 (C)
			4 (D)
Subtype			52 (inattentive)
			48 (combined)

Two different professional teams conducted pre- and post-treatment assessments, so the second team was blinded, i.e., had no knowledge of the initial neuropsychological metrics or current presentation of the disorder (predominantly inattentive or combined), as were the professionals placing children in treatment groups and the therapists conducting the CBT intervention. These three measures ensured the study was blind.

Groups were arranged depending on availability of children’s parents/guardians and timed to avoid clashing with their school timetable.

All procedures for this study were approved by the Research Ethics Committee at Universidade Federal de São Paulo (CAAE: 00568612.3.0000.5505). Parents provided written consent as well the informed assent for all children.

### Procedures

#### Intervention

All groups were medicated with long-term methylphenidate (Ritalin LA^®^, 20 mg). Dosage was standardized to a single dose administered after breakfast every day for 18 weeks. This procedure was introduced after a 2-week adaptation period using short-term methylphenidate (Ritalin^®^, 10 mg) in twice-daily doses of 5 mg after breakfast and after lunch for the first week, and 10 mg after breakfast and after lunch in the second week. Participants were regularly asked to attend medical consultation.

Cognitive behavioral therapy treatment groups attended 20 weekly sessions lasting 2 h each. All sessions were structured to follow the same routine in which the first stage (about 40 min) was used for parent and caregiver orientation and training to manage ADHD behavioral problems in the home setting. The second stage (approximately 80 min) with the children, usually started by recording mood, followed by a homework review. The TE system was introduced and tokens given out in session 5. The next step was the session’s main activity, namely: problem-solving technique, self-instruction, planning, training social skills, etc. Afterward, a related homework task was suggested. All sessions ended with a self-assessment of behavior during the session on a scale of 0–10 while therapists evaluated the behavior of each child.

#### Token-Economy Technique

**Figure 1:**
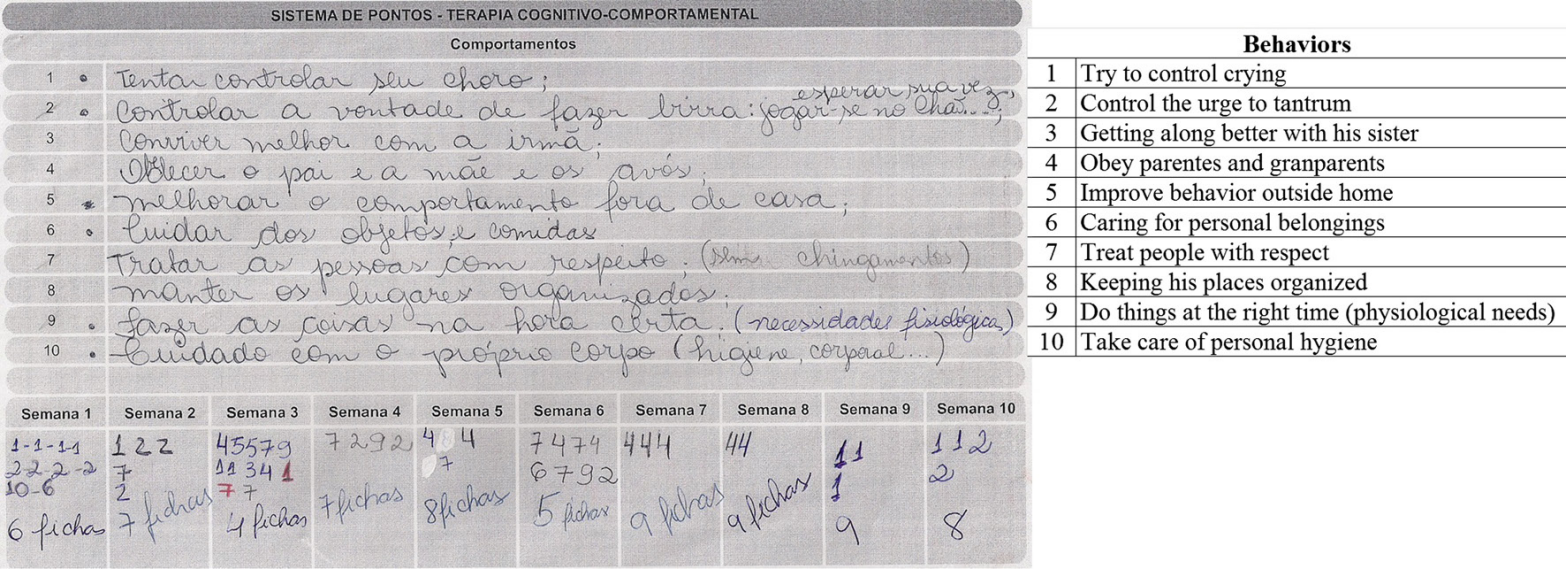
**Behaviors listed by children and their parentes**.

Our analysis was based on TE system target behaviors with positive contingency reinforcement used in this intervention program, which was introduced as of session 5. To conduct this procedure with the help of therapists, parents and children were asked to list children’s behaviors that were inappropriate in that they caused functional impairment due to negative consequences for the personal or social lives of the children themselves and their families. The ten most problematic behaviors from these lists were separated together with families and then rewritten on a card with positive phrases, i.e., what children should do or not do, and what they should avoid. Each behavior was given a number to be monitored over a 10-week period, as shown in Figure 1.

During the week, parents recorded inappropriate behaviors immediately after they took place, having been instructed to record the number of times corresponding to each of them. After recording an event, parents were to show the child how they should act and this procedure was carried out each time the child showed inappropriate behavior.

At weekly CBT sessions, behaviors were counted and children rewarded with a token for non-occurrences. Each child was given a box to keep their tokens in and told they would be exchanged for prizes after 10 sessions. At this point in the sessions, the aim was to ascertain frequency of behaviors and develop strategies for improving with children and parents. Rewards were used as positive reinforcement for appropriate behavior. In addition to tokens for correct behaviors during the week, there were also rewards unrelated to acquiring objects. Over the 10-week period, parents were shown new repertoires for mostly social-type rewards such as praise, or extra time for an activity the children liked, or having a friend over to play, or family games or going out on leisure activities. During sessions, therapists aimed to reinforce through praise. The children involved also learned to praise improvements shown by other members of the group on some occasions. After 10 weeks using this technique, points were counted and each child rewarded with an object such as a case or coloring crayons or pens depending on their age. In addition, each family individually gave rewards such as trips or leisure activities their child had wanted but had not been offered in the 10-week period.

### Statistical Analysis

Token economy system forms were used to analyze improvement (less frequent problem behavior). Behaviors were grouped in inferences of themes using content analysis technique to categorize text units (words or phrases, in this case behaviors of participating children), inferring an expression representing them or categories by analogous regrouping (34, 35). Content analysis technique consists of three main stages: (1) pre-analysis – organizing materials to systematize initial ideas, demarcating what will be used, formulating hypotheses and goals, and finally compiling indicators to be analyzed; (2) exploration of material – categories are defined by coding and identifying in recording units for the categorizing and frequency counts; (3) results are processed for interpretation (35).

This study used pre-analysis forms as shown in Figure 1 with descriptions of target behaviors listed by children and their parents. For exploring material, behavioral categories were created based on similarities and data from recording units were coded. Two specialists in CBT and neuropsychology classified behaviors separately. A third specialist (judge) recategorized any discrepancies between the first two for the final version (see supplementary material for classes of behavior generated for this analysis).

The last stage of processing results and interpretation compiled the frequency of each behavior. To analyze variations in frequencies of behavioral categories over the weeks (time), we used generalized estimating equations (GHGs) with Poisson distribution for counting data and first-order autoregressive correlation structure. Despite its being a discrete variable (0–10), time (evaluation week) was inserted into the model as a continuous variable. Interactions were analyzed for time, gender, age, IQ, and ADHD subtype. The significance level was *p* < 0.05.

## Results

The sample consisted of 25 children with ADHD, 19 boys, mean age 10.11 (SD 1.79), average IQ 108.20 (SD 12.27), 13 inattentive subtype and 12 combined subtype.

In relation to records of categorized behavioral incidents, not all children were assessed for all categories since many of them had 0 incidents recorded. On average, each child was allocated in five categories.

No statistical model could be applied to the inattention category since it contained only four children. This category of behavior was observed in 1 male (week 1), and in one male and two females (weeks 2 and 3), but then disappeared in the following weeks (5–10).

Table 2 compares means and SD for frequency of behaviors presented in the first and last intervention weeks. Briefly, the analysis showed that a statistically significant time effect (evaluation week) for seven categories: impulsiveness, hyperactivity, disorganization, disobeying rules and routine, poor self-care, easily frustrated and antisocial behaviors. Verbal/physical aggression, compulsive behaviors, and lack of initiative and execution categories did not show significant effects (see Table 3 for detailed description of the statistical analyses).

**Table 2:** **Mean and SD by behavioral category in intervention weeks 1 and 10**.

Category	*N*	Gender	Mean (SD) week 1	Mean (SD) week 10
Impulsiveness	15	10 boys; 5 girls	2.67 (8.16)	0.80 (2.04)
Hyperactivity	5	3 boys; 2 girls	1.60 (1.95)	0 (0)
Disorganization	22	16 boys; 6 girls	1.00 (1.38)	0.32 (0.72)
Disobeying rules and routine	24	19 boys; 5 girls	4.21 (4.55)	0.96 (1.37)
Poor self-care	13	9 boys; 4 girls	1.31 (2.36)	0.62 (1.94)
Verbal/physical aggression	20	16 boys; 4 girls	0.60 (1.50)	0.20 (0.70)
Easily frustrated	8	6 boys; 2 girls	3.00 (4.17)	1.13 (2.10)
Compulsive behaviors	4	2 boys; 2 girls	1.50 (2.38)	0.75 (0.96)
Antisocial behavior	12	11 boys; 1 girl	1.08 (1.78)	0.08 (0.29)
Lack of initiative and execution	13	9 boys; 4 girls	0.85 (1.68)	0.08 (0.28)

**N*, per category (same children and parents reporting at week 1 and week 10)*.

**Table 3:** **Statistical model fit**.

Category	QICC	*B*	SE	Wald Chi-square	*p*-value
Impulsiveness	743.22	−0.139	0.028	25.40	0.001
Hyperactivity	54.76	−0.316	0.063	25.27	0.001
Disorganization	682.29	−0.104	0.021	24.29	0.001
Disobeying rules and routine	873.57	−0.168	0.024	47.12	0.001
Poor self-care	301.64	−0.122	0.063	3.79	0.05
Verbal/physical aggression	396.14	−0.105	0.067	2.50	0.114
Easily frustrated	314.37	−0.134	0.050	7.25	0.007
Compulsive behaviors	78.08	0.047	0.029	2.66	0.103
Antisocial behavior	119.40	−0.300	0.092	10.66	0.001
Lack of initiative and execution	274.75	−0.081	0.082	0.98	0.323

*QICC, corrected quasi likelihood under independence model criterion*.

**B*, betas non-adjusted (time effect); see text to Betas adjusted for gender, age, IQ*.

**Figure 2:**
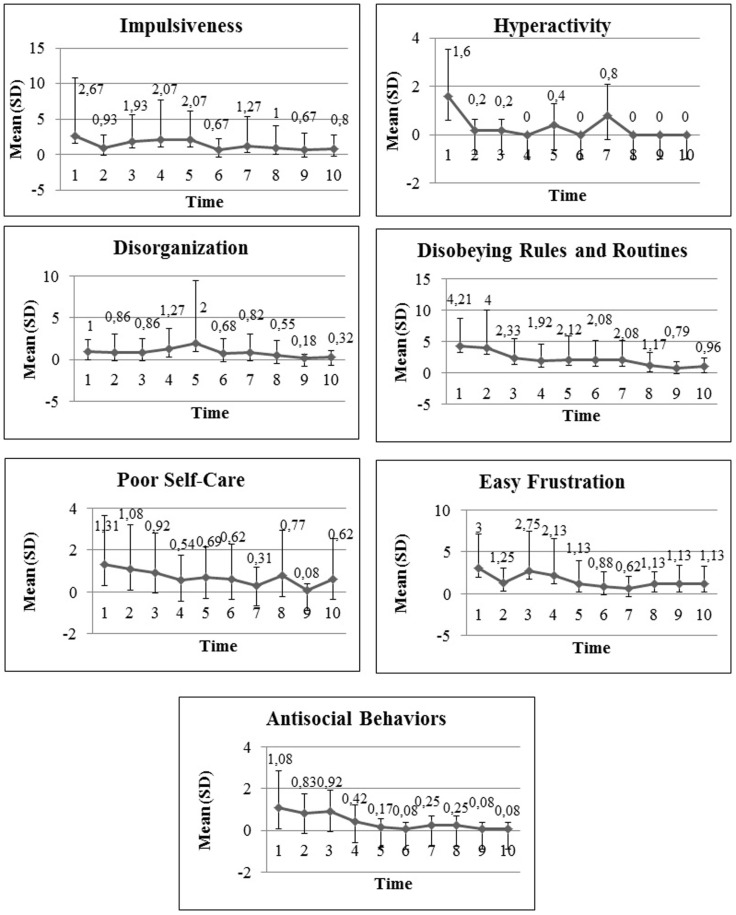
**Mean and SD for significant over the 10 weeks of treatment for significant categories**.

Figure 2 shows mean and SD over the 10 weeks of treatment for categories showing significant effects.

For impulsivity, time effect was statistically significant (*B* = −0.139, SD = 0.028, *p* = 0.001), thus showing reduced frequency of impulsive behaviors. Interaction effects were not significant.

In relation to hyperactivity, the number of children assessed was very small so covariate analysis was not applicable. In the model without covariates, time effect was significant (*B* = −0.316, SD = 0.063, *p* = 0.001) showing reduced frequency of behaviors in this category over the 10-week period.

Although some variations were observed in average frequencies in the disorganization category, there was a reduction of their frequency over the 10 week period (*B* = −0.104, SD = 0.021, *p* = 0.001). There was an age effect (*B* = 0.614 SD = 0.2289, *p* = 0.007), with older children showing higher average frequency of behavior of this category.

There was a significant reduction over time in the disobeying rules and routine category (*B* = −0.168, SD = 0.024, *p* = 0.001). Covariate analysis showed a time–gender interaction effect (*B* = 0.165, SD = 0.026, *p* = 0.001).

In the poor self-care category, time was significant (*B* = −0.122, SD = 0.063, *p* = 0.05). The covariate model showed significant time–gender interaction (*B* = 0.070, SD = 0.020, *p* = 0.001) and time–subtype interaction (*B* = 0.171, SD = 0.047, *p* = 0.001). The inattentive subtype showed higher frequency of behaviors between week 1 [inattentive *M* = 0.69 (1.93); combined *M* = 0.66 (1.72)] and week 10 [(inattentive *M* = 0.53 *M* (1.94); combined *M* = 0.08 (0.28)].

In the physical or verbal aggressiveness category, the frequency reduction over the 10 weeks was not statistically significant (*B* = −0.105, SD = 0.067, *p* = 0.114). The covariate model showed significant time–gender interaction (*B* = 0.774, SD = 0.058, *p* = 0.001) and between time and subtype (*B* = −0.393, SD = 0.098, *p* = 0.001) showing that boys and girls develop differently do combinations and inattentive behaviors.

The easily frustrated category showed a significant time effect (*B* = −0.134, SD = 0.050, *p* = 0.007). The complete covariate analysis model did not converge.

In the compulsive behaviors category, there was no reduction in frequency of behaviors over time (*B* = 0.047, SD = 0.029, *p* = 0.103). The complete covariate analysis model did not converge.

In the antisocial behavior category, time effect was significant (*B* = −0.300, SD = 0.092, *p* = 0.001). There was time–gender interaction (*B* = 0.603, SD = 0.152, *p* = 0.001) and time-IQ effect (*B* = −0.017, SD = 0.006, *p* = 0.005), showing that these two variables interfere with the time effect.

Finally, for “lack of initiative” and execution, there was no significant time effect (*B* = −0.081, SD = 0.082, *p* = 0.323), but there was time–age (*B* = 0.046; SD = 0.0215, *p* = 0.031) and time–gender interaction (*B* = 0.359, SD = 0.0722, *p* = 0.001).

## Discussion

The TE strategy has been used for interventions with children with ADHD in family, academic, and sports contexts (19–24, 27, 28). More specifically, this technique may use either positive contingency reinforcement rewarding appropriate behavior (token, praise, social prizes), or negative reinforcement in the form of response cost by withdrawing a token, for example. Given the importance of reducing inappropriate behavior using the TE, this study evaluated the dysfunctional behavior reported by parents and children. This enabled us to treat children’s difficulties more generally rather than just the three ADHD symptoms.

In relation to the behaviors analyzed, we found there was a significant intervention time effect for seven of the eleven categories analyzed: impulsiveness, hyperactivity, disorganization, disobeying routine, poor self-care, easily frustrated, and antisocial behaviors.

The inattention category frequency was very low, probably was due to the small number of children with this behavior. During the time spent setting up the TE technique with parents and children, reports of inattentive behaviors were overridden by other behaviors that were seen as more problematic from the family’s point of view at that time, such as disruptive behaviors and problems with school activities, organization, task fulfillment, and others.

In relation to compulsive behaviors, there were fewer reports, although this type of behavior translates signs of anxiety and comorbidity is quite frequent in ADHD cases (17%), while specific complaints reported in this category were more related to behavior such as fidgeting with finger nails or skin (36).

No time effect was found in the verbal and physical aggression category either. Although parents have often reported this as a frequent problem, data from this study showed a low average frequency for this behavior in the first week with little fluctuation over the 10-week period. In relation to time–gender interaction, the sample contained few girls in this category, so this effect may be related to type I statistical error. However, note that all the children were already using medication 5 weeks before we started counting frequency of behaviors. Studies comparing the effect of pharmacological treatment for children with ADHD show better control of aggressive behavior shortly after starting medication (6, 14).

Concerning to the lack of initiative and execution category, no time effect was found, as well. This category includes behaviors related to academic or domestic tasks (classroom tasks, writing homework in a diary and doing it, and starting daily routine tasks without being asked). In this case, it is important to note that participants of this sample were from low-income communities, in which parents work full-time and many children are left alone, with parents often not checking on homework or academic routine at school. Therefore, parents’ reports on the frequency of failure to execute tasks may have been adversely affected. However, the literature shows that other interventions are more effective for this type of behavior, such as combining response cost with TE technique and incremental self-regulation interventions in which children are taught to monitor their own behavior while raising level of performance on tasks (20).

It was found a time–age interaction effect in the disorganization category, showing average frequency of behavior rising with age in these categories. Some researchers have argued that older children have to cope with more academic and social demands, and older ADHD children show higher frequency of behaviors related to difficulty organizing due to increased demand (21).

In relation to the poor self-care category, the highest frequency of this behavior was shown by patients in the inattentive subtype group, with its average frequency remaining higher throughout the 10-week intervention. This category includes behaviors related to keeping routines and assiduously eating at meal times, washing/bathing, and brushing teeth. This finding suggests that even when medicated, inattentive subtype children tend to experience more difficulty engaging and keeping up with routine or highly detailed tasks, especially when they are not supervised on a daily basis, thus corroborating findings in the literature concerning differences between ADHD subtypes (2). In these cases, there are other interventions that could be of more help, such as self-regulation strategies teaching children to assess their own behavior at regular intervals using a Likert scale (20).

It was found a time effect for disruptive behaviors: impulsivity, hyperactivity, disobeying rules and routine, easily frustrated, and antisocial behavior. Behavioral interventions based on consequential strategies, such as the TE technique, helped develop self-control and improve disruptive behaviors; therefore this technique is quite efficacious specifically for these behavioral categories. These results are corroborated by findings in the literature on response to intervention in these type of behavior in children and adolescents (20–22).

One particular difficulty was noted with the implementation of the TE technique in this group. At the very beginning of the process, all participants (children and parents) were taught how to identify the target behaviors and how to register them on the form. However, in the first week meeting, parents reported they had doubts about the behavioral counting, which could generate omissions or excessive counting. So, an additional round of register training was conducted, and the doubts were no longer an issue on the following weeks. It is fact that the parents doubts may have generated some less reliable behavioral counting on the first week. However, it is possible to consider that the quantitative data are reliable, since the problems were solved on the first week, and the statistical analysis conducted was based on the behavior counting during all weeks, and not only on the comparison between the two measures of pre- and post-intervention. Also, improving the parents’ ability to identify correctly the target behaviors is an inherent part of the technique, and that takes practice.

In general, studies that specifically used the TE technique for ADHD patients have reported significantly improved behavior on comparing pre- and post-treatment data, thus corroborating our own findings (20–24, 27, 28). However, since the above studies were unable to specify which behaviors showed most improvement, their data cannot be directly compared with our own findings. In terms of behavioral modification, it is important to systematically analyze functional difficulties of patients with ADHD and not just measure reduced average frequency of behaviors emitted. This type of analysis may help make decisions concerning treatment in the case of medication (dosage, contraindications) or even for a family that prefers behavioral treatment alone.

## Conclusion and Limitations

This study showed that using TE technique as part of CBT effectively diminished externalizing (impulsivity, hyperactivity, disrupting routine, low frustration tolerance, and antisocial behavior) and internalizing behaviors (poor self-care and disorganization). This technique selects target behaviors and specific criteria to reach behavioral targets (26). The efficacy of using CBT to improve specific behaviors has to be shown with more use of evidence-based practices.

One limitation of the study was the small sample, taken from just one city in Brazil, and thus may not generalize to other countries. Other important points for future research would be to analyze data from a larger sample taken from other health services and communities around the country, as well as to control for demographic characteristics (such as mean income or type of school). Another limitation was not having a control group to compare the frequency of dysfunctional behaviors. Subtypes also were limited to inattentive and combined while the hyperactive subtype alone was not analyzed, since the frequency of some behaviors – particularly inattention – was too low for data to be statistically analyzed, as was the case for other behaviors that showed no significant effect.

Future studies should examine effects in other populations and compare different types of intervention, such as individual or group therapy, or different medication doses/adjustments and control group. Larger samples may enhance analyses of the presence of these behaviors and the use of token economy techniques to reduce their occurrence.

## Conflict of Interest Statement

The authors declare that the research was conducted in the absence of any commercial or financial relationships that could be construed as a potential conflict of interest.
