# Supplementary material for: Use of Cognitive Behavioral Therapy and Token Economy to Alleviate Dysfunctional Behavior in Children with Attention-Deficit Hyperactivity Disorder
Source: Front Psychiatry. 2015 Nov 25;6:167. doi: 10.3389/fpsyt.2015.00167 (PMC4659172; doi:10.3389/fpsyt.2015.00167)
Supplement: Supplementary file 1 [file table_1.docx]

***Supplementary Material***

**Use of cognitive behavioral therapy and token economy to alleviate dysfunctional behavior in children with attention-deficit hyperactivity disorder**

Luzia Flavia Coelho^1^, Deise Lima Fernandes Barbosa^1^, Sueli Rizzutti^1^, Mauro Muszkat^1^, Orlando Francisco Amodeo Bueno^1^, Monica Carolina Miranda^1^*.

^1^ Psychobiology Department, Universidade Federal de São Paulo, São Paulo, Brazil

*Correspondence: Dr. Monica C Miranda

Psychobiology Department - UNIFESP

Rua Embau, 54, São Paulo – SP, Zip Code 040390-060, Brasil.

mirandambr@yahoo.com.br

## Supplementary Tables 1 - Behavioral Categories

| Category | Definition and application | Examples of behavior  (mentioned by mothers) |
| --- | --- | --- |
| Inattention | Difficulty selecting and keeping focus of attention on daily tasks. | Paying more attention; paying attention in class. |
| Impulsiveness | Interrupting; not waiting; intruding; giving over hasty answers. | Speaking at the right time without shouting; patiently listening to mother. |
| Hyperactivity | Fidgeting; getting up at inappropriate times; climbing inappropriate places; running around too much. | Try to remain seated for activities; avoid chatting in class. |
| Disorganization | Difficulty keeping objects and environments organized and clean, getting behind with tasks. | Keep personal belongings organized; school and toys; checking items before going to school; tidying clothes and school supplies. |
| Disobeying rules and routine | Challenging; opposition and non-compliance with rules; instructions and established routine (times, tasks). | Washing/bathing at the right time; going to sleep at the agreed time; doing homework. |
| Poor self-care | Difficulty keeping to healthy habits; eating properly; hygiene and appearance (taking medications; brushing teeth, washing/bathing clothing). | Eating lunch even when not very hungry; washing/bathing and brushing teeth every day |
| Verbal/physical aggression | Name calling; swearing; shouts; provocations; arrogant behavior; physical aggression of any kind. | Avoid fighting and provoking sibling; get along with school friends; avoid talking back to parents.  Being aggressive to others |
| low toleration of frustration | Showing discontent disproportionate to situation ("tantrum"). | Complain less; try to control crying.  Difficulty controlling behavior without directing aggression at others |
| Compulsive behaviors | Nail biting; fingers; poke wounds; compulsive eating. | Nail biting; pinching skin on fingers. |
| Antisocial behavior | Telling lies; destroying objects; selfish attitudes; theft. | Treating dog well; always tell the truth; take responsibility for what you did, behaviors related to conduct problems. |
| Lack of initiative and execution | Difficulty starting or executing tasks properly. | Remember to start school work; start and conclude tasks. |
